# Supplementary material for: Determination of Heavy Metal Ions in Infant Milk Powder Using a Nanoporous Carbon Modified Disposable Sensor
Source: Foods. 2023 Feb 7;12(4):730. doi: 10.3390/foods12040730 (PMC9956227; doi:10.3390/foods12040730)
Supplement: Supplementary file 1 [file foods-12-00730-s001.zip › foods-2176544-supplementary.pdf]

# Supporting Information

**Table S1.** Comparison of HMIs detection by electrochemical methods and other widely used techniques.

| | | Principle | Characteristics | References |
|---|---|---|---|---|
| Spectroscopy | Atomic Absorption Spectroscopy | The absorption degree of gaseous ground state atoms to characteristic spectral lines is measured. | High sensitivity, low detection limit, little background interference. However, the pretreatment is complicated, the equipment is large and the operation is tedious. | [1-3] |
| | Atomic Emission Spectroscopy | Qualitative and quantitative analysis is carried out by measuring the characteristic spectrum of the electron transition in the outer layer of the atom under the state of electric or thermal excitation. | High sensitivity, good selectivity, fast detection speed. But the equipment is complicated and the error is large. | |
| | Atomic Fluorescence Spectrometry | The element under test is determined by measuring the emission intensity of fluorescence produced by atomic vapor of the element under radiation excitation. | The spectrum line is simple and less interference, and the linear range is wide. But the application elements are limited. | |
| Mass Spectrometry | Inductively Coupled Plasma Mass Spectrometry | The inductively coupled plasma is combined with mass spectrum, the sample is vaporized by inductively coupled plasma, the metal to be measured is separated, and then the mass spectrum is used for determination. | It can be used for qualitative analysis, semi-quantitative analysis and quantitative analysis, and can be used for the determination of various elements and isotopes at the same time. The detection limit is low. But they are expensive and vulnerable to contamination. | [4, 5] |
| Chromatography | High Performance Liquid Chromatography | Trace metal ions form stable colored complexes with organic reagents, which are separated by liquid chromatography and detected by UV-vis detector. | Simultaneous determination of multiple elements can be realized. However, the selection of complex reagents is limited, which brings limitations to the wide application of liquid chromatography. | [6] |
| Biochemical technology | Enzyme Inhibition | The toxicity of heavy metal ions can be used to reduce the enzyme activity after the combination with enzymes, the color, electrical conductivity, absorbance and other signals change, thus reflecting the type or content of heavy metals. | The detection speed is fast and the operation is simple. But the enzyme type is limited and the specificity is poor. | [7-11] |
| | Immunoassay | The specific reaction of heavy metal antigen and monoclonal antibody was used for detection. | Strong specificity. But the cost is high and it is impossible to detect multiple elements at the same time. | |
| Electrochemical method (DPASV, SWASV, LSASV) | | The electrochemical activity of the test substance is used and the electrical signal is transformed to realize qualitative and quantitative detection. | High sensitivity, fast detection speed, the ability to measure a variety of substances at the same time, easy to operate, portable instrument. | [12-15] |

DPASV: differential pulse anodic stripping voltammetry; SWASV: square wave anodic stripping voltammetry; LSASV linear sweep anoding stripping voltammetry.

**Table S2.** The main ingredients and nutrients of milk powder from five different brands.

| Brand | Main ingredients | Nutrients |
|---|---|---|
| 1 | Raw milk, lactose, edible plant blend oil, desalted whey powder, whey protein powder, walnut oil, vitamin A, vitamin D, vitamin E, vitamin K, vitamin B6, nicotinic acid, folic acid, pantothenic acid, vitamin C, biotin, potassium chloride, copper sulfate, magnesium sulfate, zinc sulfate, sodium selenite, inositol, taurine. | Energy, protein, fat (linoleic acid, $\alpha$-linolenic acid), carbohydrates, vitamins, minerals. |
| 2 | Lactose, whey protein concentrate, skim milk powder, edible plant blend oil, galactose oligosaccharide, minerals (calcium carbonate, magnesium hydrogen phosphate, ferrous sulfate, copper sulfate), vitamins (L-sodium ascorbate, inositol, palmitate vitamin A, nicotinamide, L-ascorbic acid, riboflavin), nucleotides, potassium hydroxide. | Energy, protein, fat (linoleic acid), carbohydrates, vitamins, minerals. |
| 3 | Raw milk, skim milk powder, desalted whey powder, lactose, edible plant blend oil, whey protein powder, casein, inositol, taurine, L-carnitine tartrate, nucleotides, phospholipids, vitamin A, vitamin D, vitamin E, vitamin K, vitamin B1, vitamin B2, vitamin B6, nicotinamide, folic acid, ferrous sulfate, sodium citrate, potassium citrate, potassium chloride. | Energy, protein, fat (linoleic acid, $\alpha$-linolenic acid), carbohydrates, vitamins, minerals. |
| 4 | Raw milk, desalted whey powder, lactose, vegetable oil, whey protein powder, walnut oil, anhydrous cream, phospholipids, vitamin A, vitamin D, vitamin E, vitamin K, vitamin B1, vitamin B6, vitamin B12, nicotinic acid, folic acid, pantothenic acid, vitamin C, copper sulfate, potassium iodate, inositol, taurine, L-carnitine. | Energy, protein, fat (linoleic acid), carbohydrates, vitamins, minerals. |
| 5 | Raw milk, desalted whey powder, edible plant blend oil, lactose, galactose oligosaccharides, whey protein concentrate, fructose oligosaccharides, calcium carbonate, magnesium chloride, potassium citrate, dipotassium hydrogen phosphate, sodium selenite, ferric pyrophosphate, zinc sulfate, copper sulfate, manganese sulfate, taurine, vitamin C, vitamin A, vitamin K, nicotinamide, pantothenic acid, inositol, choline. | Energy, protein, fat (linoleic acid, $\alpha$-linolenic acid), carbohydrates, vitamins, minerals. |

**Table S3.** Comparison of the detection performance of Pb(II) and Cd(II) between SPE/NPC and the reported methods.

| Electrochemical method | Electrode | Linear range (μg $L^{-1}$) | | LOD (μg $L^{-1}$) | | References |
|---|---|---|---|---|---|---|
| | | Cd(II) | Pb(II) | Cd(II) | Pb(II) | |
| DPASV | GC/Nafion-G | 1.5-30 | 0.5-50 | 0.02 | 0.02 | [16] |
| DPASV | GC/Nafion-G@MWCNTs | 0.5-30 | 0.5-30 | 0.1 | 0.2 | [17] |
| SWV | SPCE/MHCS-Nafion | 2-200 | 2-200 | 1.63 | 1.37 | [18] |
| SWV | GC/3DGO-Py | 5-400 | / | 3.6 | / | [19] |
| SWV | GC/SBA-15/L-cys | 5-80 | 5-80 | 0.22 | 0.36 | [20] |
| DPASV | ITO/p-PDMS@MSF | 30-900 | 4-1500 | 2 | 4 | [21] |
| **SWV** | **SPE/NPC** | **5-70** | **1-60** | **1.67** | **0.1** | **This work** |

LOD: limit of detection; DPASV: differential pulse anodic stripping voltammetry; GC: glassy carbon; G: Graphene; MWCNT: multi-walled carbon nanotubes; SWV: square wave voltammetry; SPCE: screen-printed carbon electrode; MHCS: microporous hollow carbon spheres; 3DGO: three-dimensional graphene oxide; Py: pyrrole; SBA-15: a kind of mesoporous silica materials; L-cys: L-cysteine; ITO: indium tin oxide; p-PDMS: plasma-triggered polydimethylsiloxane; MSF: mesoporous silica-nanochannel films; SPE: screen-printed electrode; NPC: nanoporous carbon.

**Table S4.** Recovery measurement for the detection of single variable HMIs in extracted infant milk powder.

| | Cd(II) | | | | | Pb(II) | | | |
|---|---|---|---|---|---|---|---|---|---|
| Sample | Added (μg L⁻¹) | ICP-MS (μg L⁻¹) | Our method (μg L⁻¹) | Recovery (%) | Sample | Added (μg L⁻¹) | ICP-MS (μg L⁻¹) | Our method (μg L⁻¹) | Recovery (%) |
| 1 | 0 | 0 | 0 | / | 1 | 0 | 0 | 0 | / |
| | 30 | 29.30±0.17 | 30.97±3.33 | 103.2 | | 30 | 27.89±0.11 | 26.16±0.89 | 87.2 |
| 2 | 0 | 0 | 0 | / | 2 | 0 | 0 | 0 | / |
| | 30 | 29.35±0.86 | 30.53±1.78 | 101.8 | | 30 | 28.65±0.20 | 27.24±1.83 | 90.8 |
| 3 | 0 | 0 | 0 | / | 3 | 0 | 0 | 0 | / |
| | 30 | 28.34±1.08 | 32.24±1.93 | 107.5 | | 30 | 26.59±0.39 | 21.89±2.06 | 73.0 |
| 4 | 0 | 0 | 0 | / | 4 | 0 | 0 | 0 | / |
| | 30 | 27.90±1.59 | 29.94±2.05 | 99.8 | | 30 | 25.75±0.28 | 21.51±2.73 | 71.7 |
| 5 | 0 | 0 | 0 | / | 5 | 0 | 0 | 0 | / |
| | 30 | 27.26±0.51 | 30.46±1.09 | 101.5 | | 30 | 27.59±0.41 | 27.82±1.85 | 92.7 |

**Figure S1. (A)** $N_2$ adsorption–desorption isotherms, **(B)** pore-size distribution of NPC.

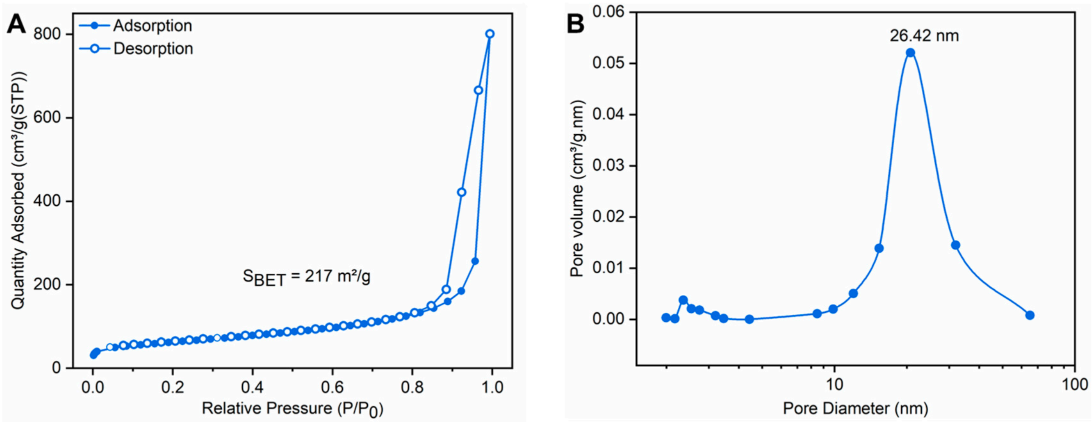

**Figure S2.** Nyquist spectra obtained with bare SPE and SPE/NPC. The impedance spectra were recorded within the range of 0.1 Hz to 100 kHz at an AC voltage of 5 mV.

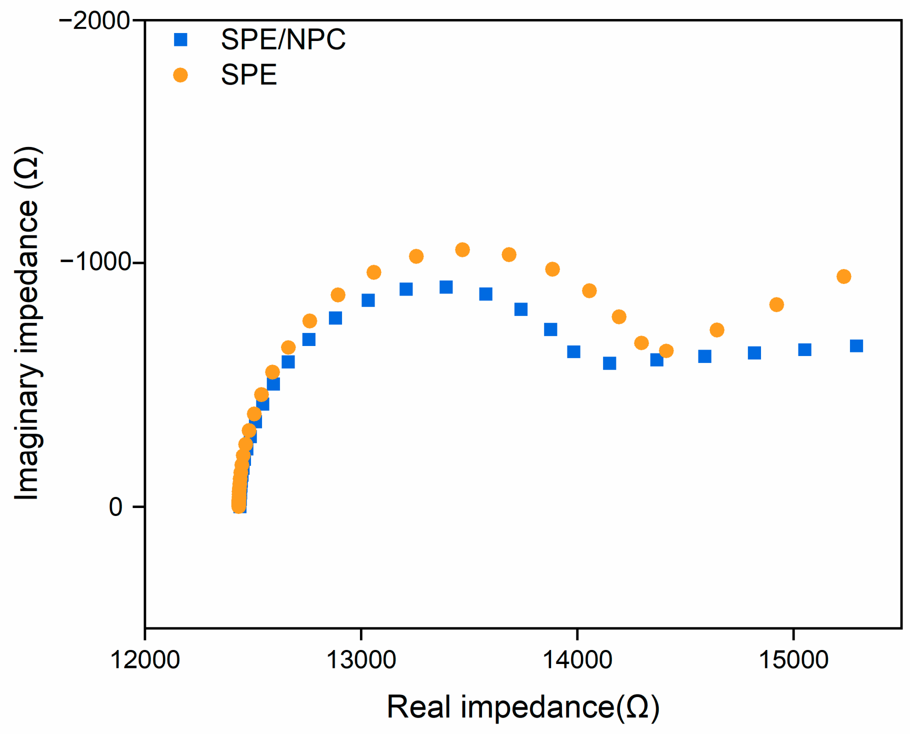

**Figure S3.** Cyclic voltammogram of bare SPE and SPE/NPC in acetate buffer solution (0.1 M, pH 4.5). Scan rate: 20 mV $s^{-1}$.

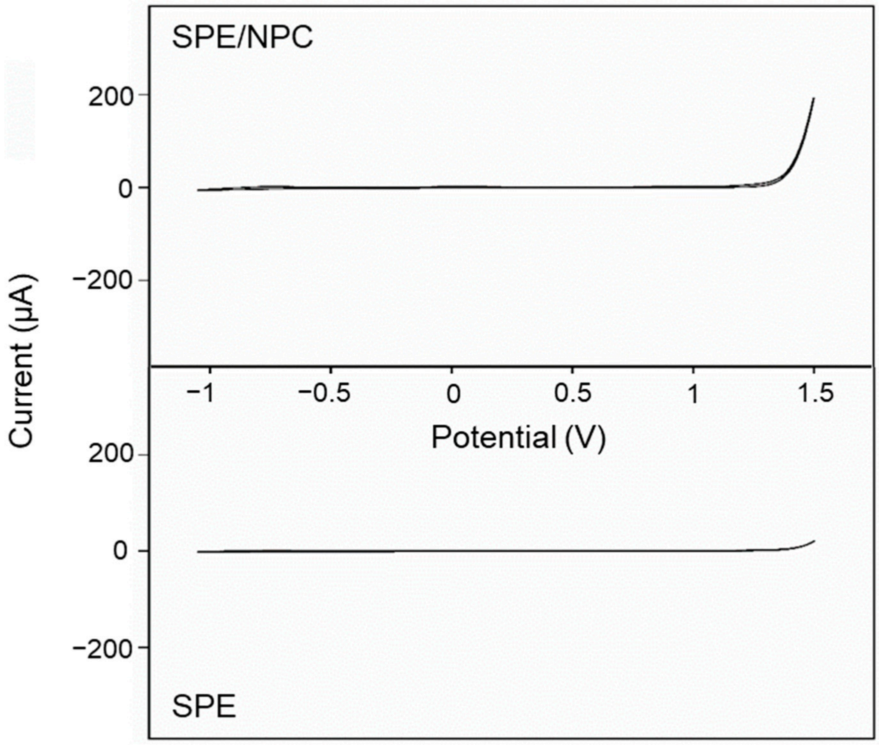

**Figure S4.** Effects of deposition volume of NPC solution on the stripping responses of SPE/NPC in the detection of Pb(II) and Cd(II) (30 μg $L^{-1}$).

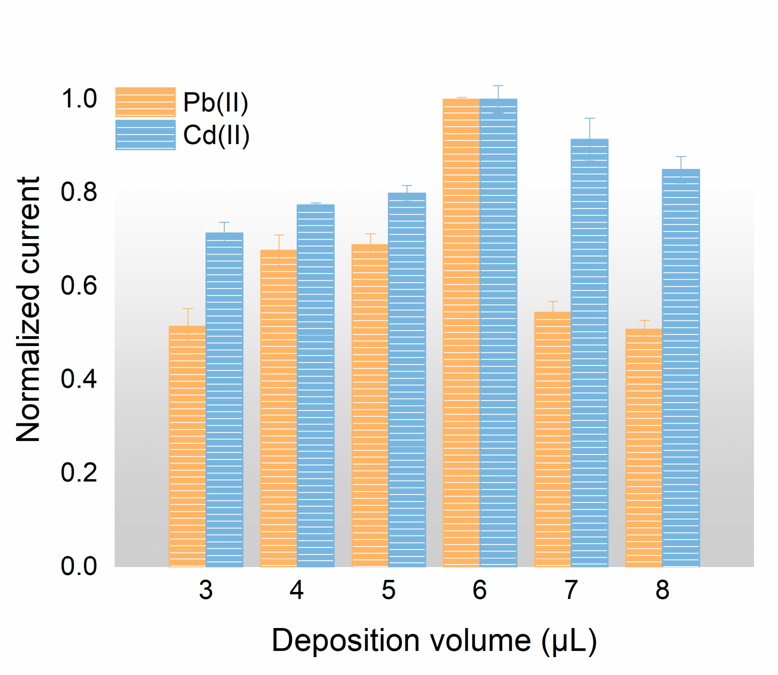

**Figure S5.** Stripping voltammograms of SPE/NPC for single variable of **(A)** Pb(II) and **(B)** Cd(II) with various concentrations in acetic acid buffer solution with 0.81 mg $L^{-1}$ Bi(III), and the calibration plots of **(C)** Pb(II) and **(D)** Cd(II).

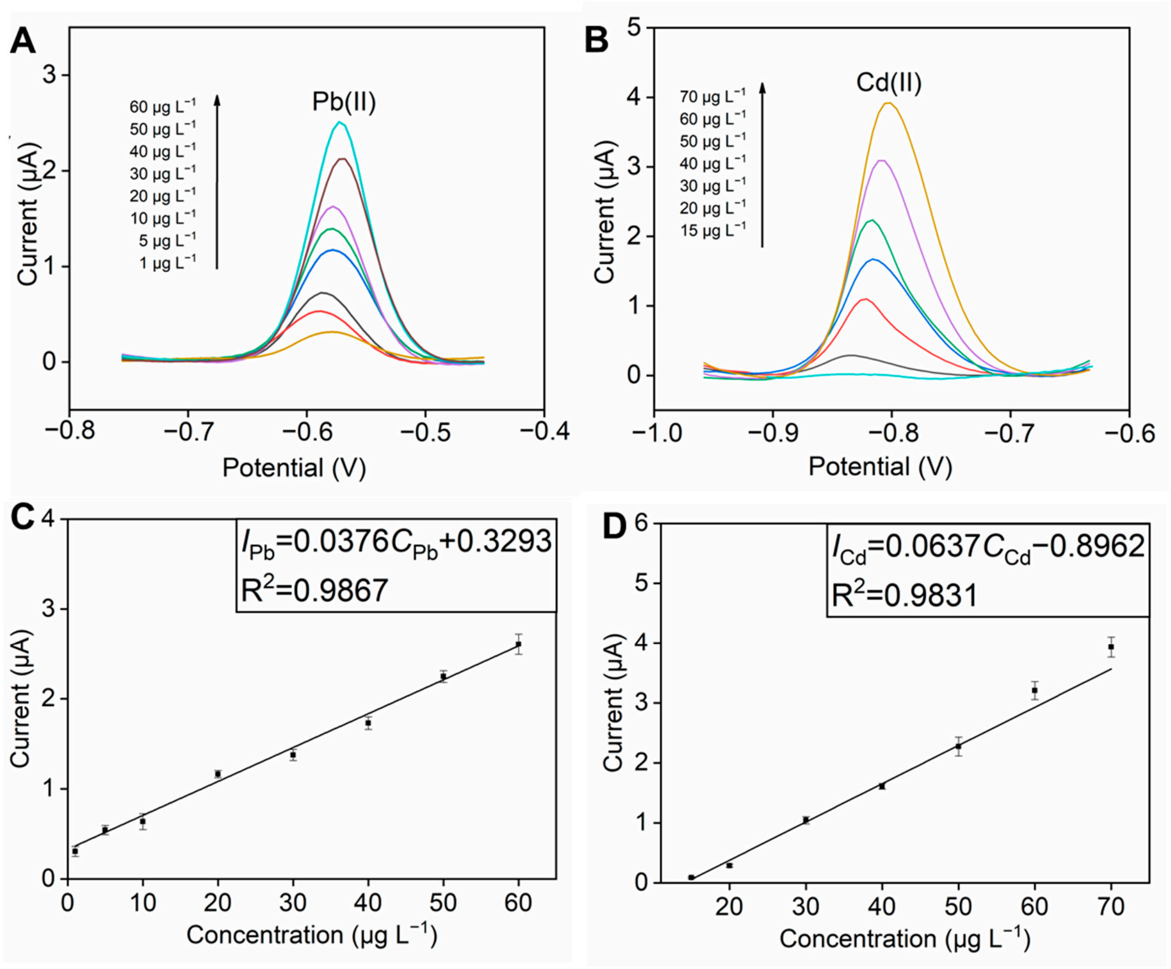

**Figure S6.** Effects of interfering substances on the stripping performance of SPE/NPC.

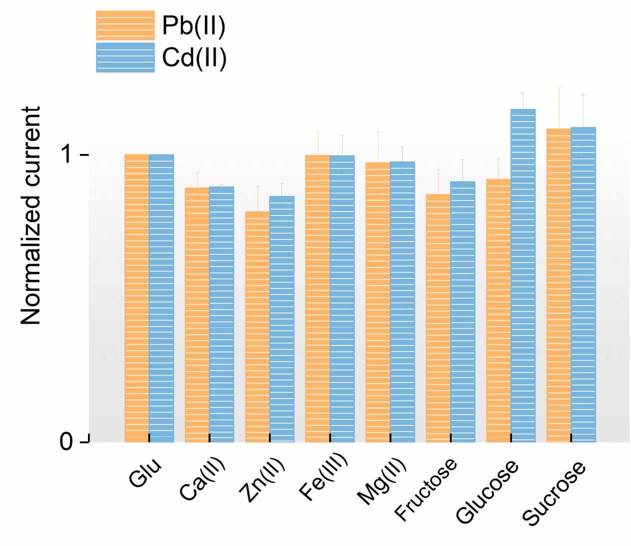

**Figure S7.** Stripping voltammograms of SPE/NPC in five infant milk powder samples before and after the addition of 30 μg $L^{-1}$ Pb(II) and Cd(II).

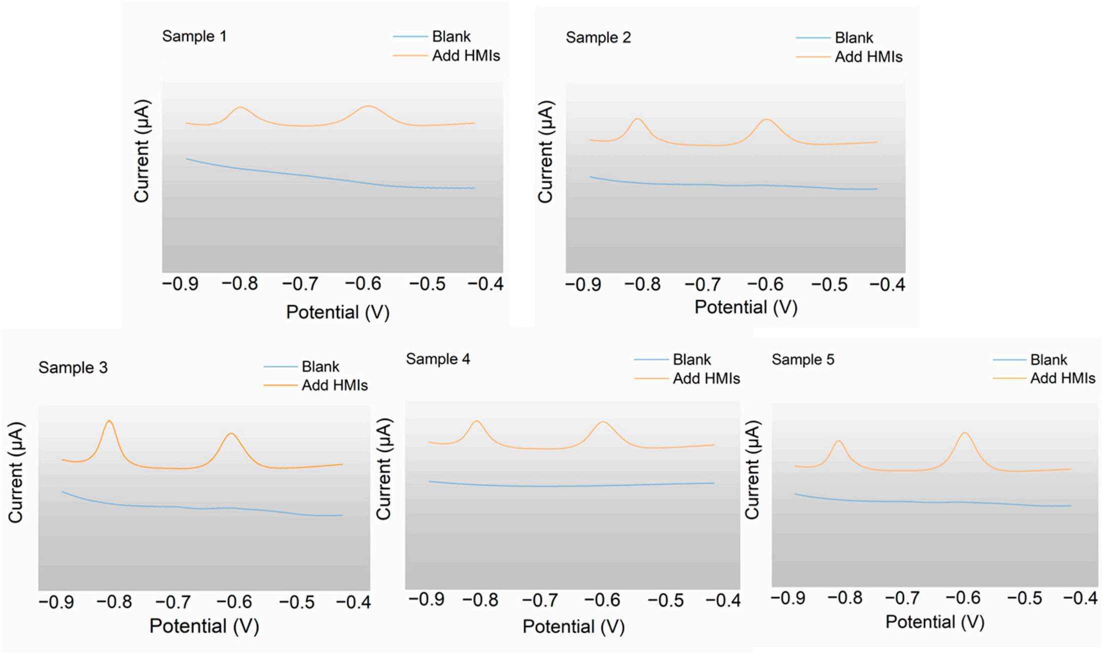


## References


1. Pan, F.; Yu, Y.; Yu, L.; Lin, H.; Wang, Y.; Zhang, L.; Pan, D.; Zhu, R. Quantitative assessment on soil concentration of heavy metal-contaminated soil with various sample pretreatment techniques and detection methods. *Environ Monit Assess*. **2020**, 192, 800.
2. Li, K.; Yang, H.; Yuan, X.; Zhang, M. Recent developments of heavy metals detection in traditional Chinese medicine by atomic spectrometry. *Microchem J*. **2021**, 160, 105726.
3. Arduini, F.; Palleschi, G., Screening and confirmatory methods for the detection of heavy metals in foods. *POPs toxic elements*. Roma Tor Vergata, Italy, **2013**, 81-109.
4. Zhang, J.; Chen, B.; Wang, H.; He, M.; Hu, B. Facile chip-based array monolithic microextraction system online coupled with icpms for fast analysis of trace heavy metals in biological samples. *Anal Chem*. **2017**, 89, 6878-6885.
5. Yu, X.; Chen, B.; He, M.; Wang, H.; Tian, S.; Hu, B. Facile design of phase separation for microfluidic droplet-based liquid phase microextraction as a front end to electrothermal vaporization-ICPMS for the analysis of trace metals in cells. *Anal Chem*. **2018**, 90, 10078-10086.
6. Burakham, R.; Srijaranai, S.; Grudpan, K. High-performance liquid chromatography with sequential injection for online precolumn derivatization of some heavy metals. *J Sep Sci*. **2007**, 30, 2614-9.
7. Wang, Y.; Zhang, C.; Liu, F. Antibody developments for metal ions and their applications. *Food Agr Immunol*. **2020**, 31, 1079-1103.
8. Ouyang, H.; Shu, Q.; Wang, W.; Wang, Z.; Yang, S.; Wang, L.; Fu, Z. An ultra-facile and label-free immunoassay strategy for detection of copper (II) utilizing chemiluminescence self-enhancement of Cu (II)-ethylenediaminetetraacetate chelate. *Biosens Bioelectron*. **2016**, 85, 157-163.
9. Date, Y.; Terakado, S.; Sasaki, K.; Aota, A.; Matsumoto, N.; Shiku, H.; Ino, K.; Watanabe, Y.; Matsue, T.; Ohmura, N. Microfluidic heavy metal immunoassay based on absorbance measurement. *Biosens Bioelectron*. **2012**, 33, 106-12.
10. Compagnone, D.; Lupu, A.S.; Ciucu, A.; Magearu, V.; Cremisini, C.; Palleschi, G. Fast Amperometric fia procedure for heavy metal detection using enzyme inhibition. *Anal Lett*. **2001**, 34, 17-27.
11. Bachan Upadhyay, L.S.; Verma, N. Enzyme inhibition based biosensors: A Review. *Anal Lett*. **2013**, 46, 225-241.

12. Wang, X.; Qi, Y.; Shen, Y.; Yuan, Y.; Zhang, L.; Zhang, C.; Sun, Y. A ratiometric electrochemical sensor for simultaneous detection of multiple heavy metal ions based on ferrocene-functionalized metal-organic framework. *Sensor Actuat B-Chem*. **2020**, 310, 127756.
13. Wang, L.; Peng, X.; Fu, H.; Huang, C.; Li, Y.; Liu, Z. Recent advances in the development of electrochemical aptasensors for detection of heavy metals in food. *Biosens Bioelectron*. **2020**, 147, 111777.
14. Nantao, L.; Diming, Z.; Qian, Z.; Yanli, L.; Jing, J.; Gang Logan, L.; Qingjun, L. Combining localized surface plasmon resonance with anodic stripping voltammetry for heavy metal ion detection. *Sensor Actuat B-Chem*. **2016**, 231, 349-356.
15. Bansod, B.; Kumar, T.; Thakur, R.; Rana, S.; Singh, I. A review on various electrochemical techniques for heavy metal ions detection with different sensing platforms. *Biosens Bioelectron*. **2017**, 94, 443-455.
16. Li, J.; Guo, S.; Zhai, Y.; Wang, E. High-sensitivity determination of lead and cadmium based on the Nafion-graphene composite film. *Anal Chim Acta*. **2009**, 649, 196-201.
17. Huang, H.; Chen, T.; Liu, X.; Ma, H. Ultrasensitive and simultaneous detection of heavy metal ions based on three-dimensional graphene-carbon nanotubes hybrid electrode materials. *Anal Chim Acta*. **2014**, 852, 45-54.
18. Niu, X.; Zhang, H.; Yu, M.; Zhao, H.; Lan, M.; Yu, C. Combination of microporous hollow carbon spheres and nafion for the individual metal-free stripping detection of $Pb^{2+}$ and $Cd^{2+}$. *Anal Sci*. **2016**, 32, 943-949.
19. Guo, X.; Cui, R.; Huang, H.; Li, Y.; Liu, B.; Wang, J.; Zhao, D.; Dong, J.; Sun, B. Insights into the role of pyrrole doped in three-dimensional graphene aerogels for electrochemical sensing Cd(II). *J Electroanal Chem*. **2020**, 871, 114323.
20. Rechotnek, F.; Follmann, H.D.M.; Silva, R. Mesoporous silica decorated with L-cysteine as active hybrid materials for electrochemical sensing of heavy metals. *J Environ Chem Eng*. **2021**, 9, 106429.
21. Li, G.; Belwal, T.; Luo, Z.; Li, Y.; Li, L.; Xu, Y.; Lin, X. Direct detection of $Pb^{2+}$ and $Cd^{2+}$ in juice and beverage samples using PDMS modified nanochannels electrochemical sensors. *Food Chem*. **2021**, 356, 129632.
